# Recent Attacks on the Uniform System

**Published:** 1916-02-12

**Authors:** 


### RECENT ATTACKS ON THE UNIFORM SYSTEM.

### Some Criticisms and Suggestions.

The Uniform System is based upon the principle that hospitals should render a regular and intelligible account of their stewardship to those who provide the means to carry on the merciful work of caring for and supplying essential treatment to the sick poor when in need of it. It is not sufficient that this account shall satisfy the ideas of what is intelligible to the mind of the person who prepared it, for methods of accountancy vary considerably; it must be in such a form that a natural or potential subscriber may apply standards to it and so consider it side by side with another account capable of the same scrutiny. Obviously, then, the account of stewardship must not apply merely to the transactions of a given period, for one institution may be passing through parlous times and yet be financially strong, while another, revealing a brilliantly successful year from the point of view of receipts, may have so little in the shape of reserves that one bad year might cripple it most seriously.

### Revision of the Uniform System.

Thus far, one would consider the position of a single system unassailable, and that the necessity for the publication of an income and expenditure account and a balance-sheet prepared on a standard plan needs no apology. Further, the position of the Uniform System is all the more secure because of the history of how the standards were attained. Here we find no autocracy; in respect of every point of importance the advice has been sought of leading representatives of those who are called upon to prepare the accounts under the system. When the counsel of these representatives has been found in experience to be faulty, revision has been made under the same popular scheme. Any system, however good, is generally capable of improvement and renovation. Revision has, therefore, been continued until a position appears to have been reached when little remains to be done except the periodical adjustment made necessary by legislation such as the National Health Act, or by temporary conditions of an unusual character such as the special financial arrangements made necessary by the present semi-military character of some of our hospitals. These are inevitable. But to consider any return to the old *laissez-aller* days is unthinkable. Yet we have within the last few weeks had before us criticisms, amateur and professional (in the accountancy sense), which contain an undercurrent of such a thought. One prominent criticism is that the accounts as published do not appear to excite the attention of the supporter of a hospital. However much the master may think the steward is doing well, it is not often that he claps him on the back and tells him so. If, on the other hand, the steward is not giving a just account the master will not be slow to speak. It is not a bad sign that subscribers make so few inquiries about the accounts, although it must not, conversely, be considered in this light if in individual cases they do. But if, in respect of hospital accounts generally, a multiplicity of inquiries were made, it would be evidence of something more than interest on the part of the individual and would tend to show that the principles of uniformity, containing the provision of essential and comparable information, had been broken down. But does the average donor understand the accounts as presented? is a question that has been put forward lately by more than one critic. We can answer in the affirmative an inquiry in similar terms, but much more to the point: Is the average giver to a hospital capable of understanding the accounts as presented if he is sufficiently interested to examine and study them with the attention that is necessary to obtain an understanding of any accounts?

### The Need of a Balance-Sheet.

From the point of view of understanding, the publication of a balance-sheet has been attacked. Subscribers differ in character and position. One hospital may derive most of its revenue in small sums from people unassociated with business, who are quite content to rely upon the executive that all is in order in respect of the financial side of the work. Another, regularly or occasionally, has to solicit large sums from business men who will apply the same habitual caution to almsgiving as they would to the operations of their business life. To the latter a balance-sheet is more than a desirability, it is a necessary. The writer can confirm this fact by at least one instance recently coming under his notice. The secretary of a hospital made an appeal to a wealthy business man, and as a result was placed in closer touch with him. The first question was as to the accounts of the previous year, and elicited the fact that there had been a substantial balance on the right side. "Then why do you ask me for help? The production of the balance-sheet at once showed that the balance on the accounts of the year previous to the good one had been against the hospital to a much greater extent, and that the lost ground had not even yet been made up. When an application is made to the Corporation of the City of London, it must be supplemented by the income and expenditure of the past three years. Here, again, spurious prosperity may be shown, which can only be explained by the production of balance-sheets. It is a truism in business circles that no accounts are complete without the clear statement of the whole financial position as shown by a balance-sheet. The chief objection to the income and expenditure account is in respect of complexity, which is due to the fine sifting of the expenditure figures to bring them within the proper headings and sub-headings; the instrument employed for this purpose is an index of classification, prepared and revised by a committee of hospital secretaries, who, of all people, should know what is the most convenient form of presenting hospital figures. There is, certainly, one difficulty in the Uniform System, and it is that part the working of which is left to the judgment of the individual accountant and cannot be described and directed in the same way as can be other features—namely, apportionment, or the division of certain items of outgoings between various headings and between in-patient and out-patient expenditure. In the papers in these columns on the King's Fund statistics this difficulty has been dealt with several times. In an article in the *Hospital Gazette*, a chartered accountant has put forward one or two sensible suggestions in respect of this point. What is the auditor's duty in reference to allocation of items of expenditure under the different headings?

We will suppose that an auditor is considering an allocation or apportionment made by the institutional secretary or accountant twelve months before. Let us say that the hospital carpenter has been supplied with a quantity of timber; some of this material will be used for making ward furniture, some for sundry repairs to fixtures in connection with the building. The accountant asks for a ruling, and the secretary directs that a certain part of the expenditure shall go to " Domestic—renewal and repair of furniture," and part to " Establishment—renewals and repairs," the proportions being decided by the amount of work done for each department. No written note is taken of the reasons for the transaction, and when the auditor wishes the matter explained twelve months later, the transaction is very difficult to recall. Hence the second suggestion of the writer referred to—that there should be a running audit. It can be successfully argued that if the auditor were in more constant attendance he would be in a better position to ratify these allocations of expenditure and accept some responsibility for the operations of the hospital accountant. He would be able also to keep a better watch upon things connected with the other side of the account.

### The Prevention of Embezzlement.

It so happens that recently one of those miserable events, which fortunately are not of frequent occurrence, has come under the notice of the public. A junior official of a hospital had been entrusted with certain financial duties which are customarily in most hospitals in the hands of the secretary, or in the very large institutions confided to a suitably paid cashier. The junior in question, by some juggling with the bank pay-ins and the passbook, succeeded in stealing a substantial sum of money. Here is an instance where an auditor, having a current knowledge of the office transactions and constant access to the books, would most probably have prevented the trouble. This leads to a suggestion which may be considered to be worthy of discussion. Why not divide the hospitals of the country into groups —one group for London, one for the Southern counties, one for the Midlands, one for Scotland, and so on. Then appoint a firm of accountants actually to keep the books of each group, and another firm, should it be deemed necessary, to audit the accounts at the end of each year? It will be required that the accountants and auditors shall possess an intimate knowledge of the Uniform System and shall confer upon its main points from time to time. Possibly this is the only means to ensure entire uniformity, and of providing an efficient means of checking any form of fraud on an important scale.

